# The efficacy of motivational counselling and SMS reminders on daily sitting time in patients with rheumatoid arthritis: a randomised controlled trial

**DOI:** 10.1136/annrheumdis-2016-210953

**Published:** 2017-06-05

**Authors:** Tanja Thomsen, Mette Aadahl, Nina Beyer, Merete Lund Hetland, Katrine Løppenthin, Julie Midtgaard, Robin Christensen, Mikkel Østergaard, Poul Jørgen Jennum, Bente Appel Esbensen

**Affiliations:** 1 Copenhagen Center for Arthritis Research, Center for Rheumatology and Spine Diseases, Centre for Head and Orthopaedics, Rigshospitalet, University of Copenhagen, Glostrup, Denmark; 2 Research Centre for Prevention and Health, The Capital Region of Denmark, Glostrup; 3 Department of Public Health, Faculty of Health and Medical Sciences, University of Copenhagen, Copenhagen, Denmark; 4 Musculoskeletal Rehabilitation Research Unit, Bispebjerg and Frederiksberg Hospitals, University of Copenhagen, Copenhagen, Denmark; 5 Department of Clinical Medicine, Faculty of Health and Medical Sciences, University of Copenhagen, Copenhagen, Denmark; 6 The DANBIO Registry, Center for Rheumatology and Spine Diseases, Centre for Head and Orthopaedics, Rigshospitalet - Glostrup, Glostrup, Denmark; 7 University Hospitals Centre for Health Research, Rigshospitalet, Copenhagen University Hospital, Copenhagen, Denmark; 8 Musculoskeletal Statistics Unit, The Parker Institute, Copenhagen University Hospital, Bispebjerg and Frederiksberg, Copenhagen, Denmark; 9 Danish Center for Sleep Medicine, Department of Clinical Neurophysiology, Rigshospitalet, University of Copenhagen, Copenhagen, Denmark

**Keywords:** multidisciplinary team-care, rheumatoid Arthritis, rehabilitation, cardiovascular Disease, patient perspective

## Abstract

**Objectives:**

The aim of this report is to investigate the efficacy of an individually tailored, theory-based behavioural intervention for reducing daily sitting time, pain and fatigue, as well as improving health-related quality of life, general self-efficacy, physical function and cardiometabolic biomarkers in patients with rheumatoid arthritis (RA).

**Methods:**

In this randomised controlled trial 150 patients with RA were randomised to an intervention or a no-intervention control group. The intervention group received three individual motivational counselling sessions and short message service or text messages aimed at reduction of sedentary behaviour during the 16-week intervention period. Primary outcome was change in daily sitting time measured objectively by ActivPAL. Secondary outcomes included change in pain, fatigue, physical function, general self-efficacy, quality of life, blood pressure, blood lipids, haemoglobin A1c, body weight, body mass index, waist circumference and waist–hip ratio.

**Results:**

75 patients were allocated to each group. Mean reduction in daily sitting time was −1.61 hours/day in the intervention versus 0.59 hours/day increase in the control group between-group difference −2.20 (95% CI −2.72 to −1.69; p<0.0001) hours/day in favour of the intervention group. Most of the secondary outcomes were also in favour of the intervention.

**Conclusion:**

An individually tailored, behavioural intervention reduced daily sitting time in patients with RA and improved patient-reported outcomes and cholesterol levels.

**Trial registration number:**

NCT01969604; Results.

## Introduction

Rheumatoid arthritis (RA) causes disability^1^ and barriers for exercise.^2^ Patients with RA have a 50%–60% increased risk of premature death from cardiovascular disease.^3^ Supplementary to the pharmacological treatment, patients are recommended to engage in moderate-to-high intensity aerobic and resistance training.^4 5^ Most patients do not meet recommended levels of moderate-to-vigorous physical activity^6^ and 71%–92% of waking hours are spent sedentarily.^7^ Sedentary behaviour is defined as sitting or reclining while awake and with low-energy expenditure.^8^ In patients with chronic disease and mobility limitations, replacing sedentary behaviour with light intensity activities may prove more achievable than solely focusing on increasing moderate-to-vigorous physical activity.^9 10^ Studies have shown that reduction of daily sitting time through intervention is possible.^11 12^ Also, improved resting blood pressure, insulin levels and plasma glucose following regular interruptions of prolonged sitting have been reported.^13^ We aimed to investigate the efficacy of an individually tailored, theory-based behavioural intervention for reducing daily sitting time, pain and fatigue, as well as improving quality of life, general self-efficacy, physical function and cardiometabolic biomarkers in patients with RA.

## Methods

We performed an observer-blinded randomised controlled trial. The protocol was reported to the Danish Data Protection Agency (711-1-08), approved by the Ethics Committee of the Capital Region of Denmark (H-2-2012-112) and registered at www.clinicaltrials.gov (NCT01969604). The Danish National Board of Health Biological Therapies (DANBIO) database^14^ was searched for potential participants. A detailed description of the methods of the trial has previously been published. See protocol and feasibility paper.^15 16^

Patients were randomised 1:1 to intervention (n=75) or control group (n=75) by computer generated random numbers in blocks of 10. Participants and project staff delivering the intervention were unblinded to the participants’ allocation status, whereas outcome assessors and the statistician were blinded to allocation. The 16-week individually tailored, behavioural intervention consisted of three motivational counselling sessions conducted by health professionals and individual short message service (SMS) or text messages aiming to increase light intensity physical activity through reduction of sedentary behaviour. Participants randomised to the control group were instructed to maintain their usual lifestyles.

The primary outcome measure was change in daily sitting time measured by an ActivPAL 3TM V.7.2.32 Activity Monitor (PAL Technologies, Glasgow, UK). The ActivPAL uses accelerometer-derived information to determine time spent sitting/lying, standing and stepping and is validated in patients with RA.^17^ The participants wore the monitor 24 hours per day for 7 days at baseline and by end of intervention, and recorded their daily sleeping time to separate sleep from waking sitting/lying time.

Secondary outcomes were changes from baseline to 16 weeks in self-reported daily sitting time at work and during leisure time and number of interruptions (‘breaks’) in daily sitting time, pain, fatigue physical function, quality of life (QoL) and general self-efficacy.^15^ Height was measured at baseline. Body weight, hip and waist circumference were additionally measured after 16-week intervention and body mass index (BMI, kg/m^2^) and waist–hip ratio were calculated. Venous blood sample were drawn. Total cholesterol, high-density lipoprotein cholesterol, low-density lipoprotein cholesterol, triglycerides, haemoglobin A1c and resting blood pressure were measured. Pharmacological treatment, duration of RA, C reactive protein, disease activity (Disease Activity Score 28), IgM rheumatoid factor and anti-cyclic citrullinated peptide (CCP) status were retrieved from DANBIO. Additional characteristics were obtained from a self-report questionnaire.^15^

Data analyses were based on the intention-to-treat population and carried out using SAS V.9.3 (SAS Institute) according to the protocol.^15^ Missing data were replaced with the value at baseline carried forward. All reported p values and 95% CIs were two sided. Unless stated otherwise, results are expressed as the difference between the group (least-squares) means and 95% CI, based on a general linear model: data were analysed using analysis of covariance with a factor for group and baseline values as covariates in the model. For dichotomous outcomes, proportions were compared based on the risk difference with 95% CIs, as well as including a Wald z test.

The trial was powered for a comparison between the participants allocated to intervention and control group, assuming that the intervention group condition would produce a reduction in daily sitting time of 50 min. Enrolling 75 patients in each group had a reasonable power (84.7%) to detect a mean difference of 50 min.^15 16^ A patient with RA from the Danish Rheumatism Association was involved in designing of the trial, including intervention and patient information.^18^

## Results

### Participants

One thousand and eight patients were screened via DANBIO, hereof 801 (79%) were invited. Telephone-based screening was conducted with 722 of these, hereof 617 (85%) were eligible. Of these, 467 declined to participate (online supplementary figure S1). Compared with those declining participation, the included patients were older (60 vs 52 years), had longer disease duration (15 vs 12 years), lower Health Assessment Questionnaire (HAQ) (0.7 vs 1.1) and more were women (81% vs 69%). Outcomes were obtained for 147 (98%) of the randomised patients.

10.1136/annrheumdis-2016-210953.supp2 Supplementary figure 1

The intervention group had higher scores on fatigue, pain, had more daily sitting time and self-reported leisure-time sitting than the control group (9.8 vs 8.8 hours and 5.3 vs 4.3 hours, respectively) (online supplementary table S1). All in the intervention group completed the counselling sessions (30–90 min) and had SMS reminders.

10.1136/annrheumdis-2016-210953.supp1 Supplementary table 1

### Primary outcome

Reductions in daily sitting time favoured the intervention group (online supplementary figure S2). Estimates of intervention effect for behavioural and patient-reported outcomes are presented in table 1. Objectively measured daily sitting time decreased in intervention group by on average 1.61 hours/day and increased in control group by 0.59 hours/day. The difference in change between groups was statistically significant in favour of intervention group (−2.20 hours/day (95% CI −2.72 to −1.69)). The decrease in daily sitting time was replaced by increased standing and stepping time with between-group differences in change of 1.52 hours/day and 0.55 hours/day, respectively.

10.1136/annrheumdis-2016-210953.supp3 Supplementary figure 2

**Table 1:** Mean changes in behavioural and patient-reported outcomes after 16 weeks

Variable	Mean change from baseline mean (95% CI)		Difference in change between groups mean (95% CI)	p Value
	Intervention group	Control group		
Daily sitting time (ActivPAL) hours/day	−1.61 (−1.97 to −1.25)	0.59 (0.24 to 0.95)	−2.20 (−2.72 to −1.69)	<0.0001
Daily standing time* (ActivPAL) hours/day	1.25 (0.82 to 1.68)	−0.27 (−0.45 to 0.78)	1.52 (1.10 to 1.95)	<0.001
Daily stepping time* (ActivPAL) hours/day	0.50 (0.26 to 0.95)	−0.05 (−0.32 to 0.64)	0.55 (0.35 to 0.74)	<0.001
Breaks up of daily sitting (ActivPAL) (number/day)	−0.47 (−3.52 to 2.57)	−1.97 (−5.02 to 1.07)	1.50 (−2.81 to 5.81)	0.49
Self-reported sitting time at work (hour/day)	−1.12 (−1.68 to −0.57)	0.005 (0.54 to 0.55)	−1.13 (−1.90 to −0.35)	0.005
Self-reported sitting time in leisure (hour/day)	−1.30 (−1.68 to −0.93)	0.15 (−0.22 to 0.53)	−1.46 (−2.00 to −0.92)	<0.0001
Physical function (HAQ)	−0.28 (−0.36 to −0.19)	0.14 (0.06 to 0.22)	−0.42 (−0.54 to −0.30)	<0.0001
Fatigue (VAS)/mm	−19.04 (−24.22 to −13.86)	7.77 (2.59 to 12.95)	−26.80 (−34.32 to −19.30)	<0.0001
Fatigue (MFI)				
General fatigue	−2.17 (−3.00 to −1.35)	1.25 (0.44 to 2.07)	−3.43 (−4.59 to −2.26)	<0.0001
Physical fatigue	−3.18 (−4.02 to −2.34)	1.34 (0.50 to 2.18)	−4.52 (−5.73 to −3.30)	<0.0001
Mental fatigue	−1.80 (−2.50 to −1.10)	0.65 (−0.05 to 1.35)	−2.46 (−3.46 to −1.46)	<0.0001
Reduced activity	−3.28 (−4.05 to −2.50)	1.60 (0.83 to 2.37)	−4.88 (−5.99 to −3.77)	<0.0001
Reduced motivation	−1.35 (−2.00 to −0.69)	1.26 (0.60 to 1.91)	−2.60 (−3.54 to −1.67)	<0.0001
Pain (VAS)/mm	−14.77 (−19.50 to −10.04)	7.59 (2.58 to 12.32)	−22.36 (−29.27 to −15.44)	<0.0001
Self-efficacy (GSES)	3.96 (2.80 to 5.12)	−2.25 (−3.41 to −1.09)	6.21 (4.54 to 7.88)	<0.0001
HR-QoL (SF-36)				
SF36-PCS	6.30 (4.33 to 8.26)	−2.58 (−4.54 to −0.61)	8.88 (6.06 to 11.69)	<0.0001
SF36-MCS	4.94 (3.42 to 6.46)	−1.83 (−3.34 to −0.32)	6.77 (4.62 to 8.92)	<0.0001

*Not an outcome measure, however, changes in daily sitting, standing and/or stepping time are interdependent, and reduced sitting time may be replaced by either standing or stepping time.

GSES, General Self-efficacy Scale; HAQ, Health Assessment Questionnaire; HR-QoL, Health-Related Quality of Life; MCS, Mental Component Scale; MFI, Multidimensional Fatigue Inventory; PCS, Physical Component Scale; SF36, 36-Item Short Form Survey Instrument; VAS, Visual Analogue Scale.

### Secondary outcomes

Statistically significant differences in favour of the intervention group were found in self-reported daily sitting time at work and during leisure time, for fatigue, pain, physical function, QoL, general self-efficacy and in total cholesterol (tables 1 and 2); also significantly greater proportions achieved clinically meaningful improvements in physical function (HAQ) (minimal clinically important difference (MCID)=0.22), fatigue (Visual Analogue Scale) (MCID=10 mm) and pain (MCID=10 mm) (table 2).^19^

**Table 2:** Proportions of participants achieving clinically important improvements in physical function, fatigue and pain with corresponding risk differences

Variable	Number (%)		Risk difference (95% CI)	p Value
	Intervention group	Control group		
Achieved 0.22 improvement in HAQ scores	38 (51)	4 (5)	46% (33% to 58%)	0.0001
Achieved 10 mm improvement on VAS for fatigue	46 (62)	10 (14)	48% (35% to 62%)	0.0001
Achieved 10 mm improvement on VAS for pain	47 (64)	9 (12)	51% (38% to 64%)	0.0001

HAQ, Health Assessment Questionnaire; VAS, Visual Analogue Scale.

For anthropometric and cardiometabolic measures, no statistically significant differences were found, but numerical differences in change were all in favour of intervention group (table 3).

**Table 3:** Mean changes in anthropometric and cardiometabolic biomarkers after 16 weeks

Variable	Mean change from baseline, mean (95% CI)		Difference in change between groups, mean (95% CI)	p Value
	Intervention group	Control group		
Weight (kg)	0.00 (−0.91 to 0.92)	0.58 (−0.34 to 1.49)	−0.58 (−1.87 to 0.72)	0.38
Waist circumference (cm)	−0.80 (−1.90 to 0.30)	0.71 (−0.39 to 1.81)	−1.51 (−3.07 to 0.05)	0.056
Waist–hip ratio	−3.03 (−4.72 to 1.35)	−1.81 (−3.50 to −0.13)	−1.22 (−3.60 to 1.16)	0.31
Body mass index (kg/m^2^)	0.02 (−0.30 to 0.32)	0.16 (−0.17 to 0.49)	−0.14 (−0.60 to 0.28)	0.46
Blood pressure (mm Hg)				
Systolic	−3.06 (−5.98 to −0.14)	−1.57 (−4.49 to 1.34)	−1.49 (−5.61 to 2.64)	0.47
Diastolic	−0.85 (−2.38 to 0.69)	−0.08 (−1.62 to 1.45)	−0.77 (−2.94 to 1.40)	0.49
Lipids (mmol/L)				
Cholesterol	−0.24 (−0.33 to −0.14)	0.13 (0.04 to 0.23)	−0.37 (−0.50 to −0.24)	<0.0001
HDL	0.06 (0.00 to 0.12)	0.00 (−0.06 to 0.05)	0.07 (−0.01 to 0.14)	0.10
LDL	−0.07 (−0.18 to 0.04)	−0.03 (−0.13 to 0.08)	−0.04 (−0.20 to 0.11)	0.61
Triglyceride	0.06 (−0.06 to 0.18)	0.06 (−0.06 to 0.17)	0.00 (−0.16 to 0.17)	0.97
HbA1c (mmol/mol)	−0.05 (−0.22 to 0.12)	0.10 (−0.07 to 0.27)	−0.15 (−0.40 to 0.09)	0.22

HbA1c, haemoglobin A1c; HDL, high-density lipoprotein cholesterol; LDL, low-density lipoprotein cholesterol.

## Discussion

Individual motivational counselling sessions during a 16-week period accompanied by individual SMS reminders reduced daily sitting time by more than 2 hours compared with the control group. Patient-reported outcomes also improved and, to a lesser extent, cardiometabolic biomarkers. Patients with RA need to manage consequences of an unpredictable disease every day, why the intervention was individualised and targeted sedentary behaviour. This whole-day approach was also targeted in a similar individually tailored, behavioural intervention aiming to reduce daily sitting time in healthy adults.^12^ That study showed a non-significant between-group difference in daily sitting time of −0.32 hours, but a statistically significant difference in waist circumference and fasting insulin levels in favour of the intervention group after 6-month intervention. Our 16-week intervention period may not be long enough to detect significant changes in other cardiometabolic biomarkers than total cholesterol. Additionally, the changes in waist circumference almost reached statistical significance, which supports the call for a longer intervention period.

The magnitude of changes in physical function, fatigue and pain was also assessed by looking at a clinical impact of the intervention on RA-related outcomes. Achievement of the MCID was consistently reached for greater proportions in the intervention group. Without neglecting the important and well-established health benefits of engaging in moderate-to-vigorous physical activity, our results indicate that patients with RA can achieve substantial health benefits by reducing sitting time. This would have implications for clinical practice and physical activity recommendations.

Strengths include the randomised controlled design, blinding of outcome assessors and objective measurements. The two groups differed in their baseline measures with respect to daily sitting time, pain and fatigue. However, we regard these differences as random occurrences.

We cannot rule out that the significant changes in cholesterol levels and self-reported clinical outcomes were reached by other pathways than through increases in low-intensity, non-exercise physical activity, for example, through healthy dietary habits.

The results may not be generalisable to all patients with RA, since those how declined participation were younger and the proportion of men was higher. The intervention may have been more appealing to women, since 81% of the included patients were women; however, up to 75% of patients with RA are women.^20^ It is also noteworthy that participants were older and had longer disease duration than non-participants. Focus on light everyday activities and individual tailoring may be particular appealing to this group of patients. Only three participants (2%) dropped out at end of intervention, which underlines the acceptability of the individually tailored intervention allowing them to set achievable goals for change in everyday activities.

In conclusion, a randomised, observer-blinded 16-week individually tailored, theory-based behavioural intervention with motivational counselling and SMS reminders reduced daily sitting time by an average of 2 hours, improved general self-efficacy, QoL, physical function, total cholesterol and reduced levels of pain and fatigue in sedentary patients with RA.
