# Supplementary figures and images for: The efficacy of motivational counselling and SMS reminders on daily sitting time in patients with rheumatoid arthritis: a randomised controlled trial
Source: Ann Rheum Dis. 2017 Jun 5;76(9):1603–6. doi: 10.1136/annrheumdis-2016-210953 (PMC5561370; doi:10.1136/annrheumdis-2016-210953)

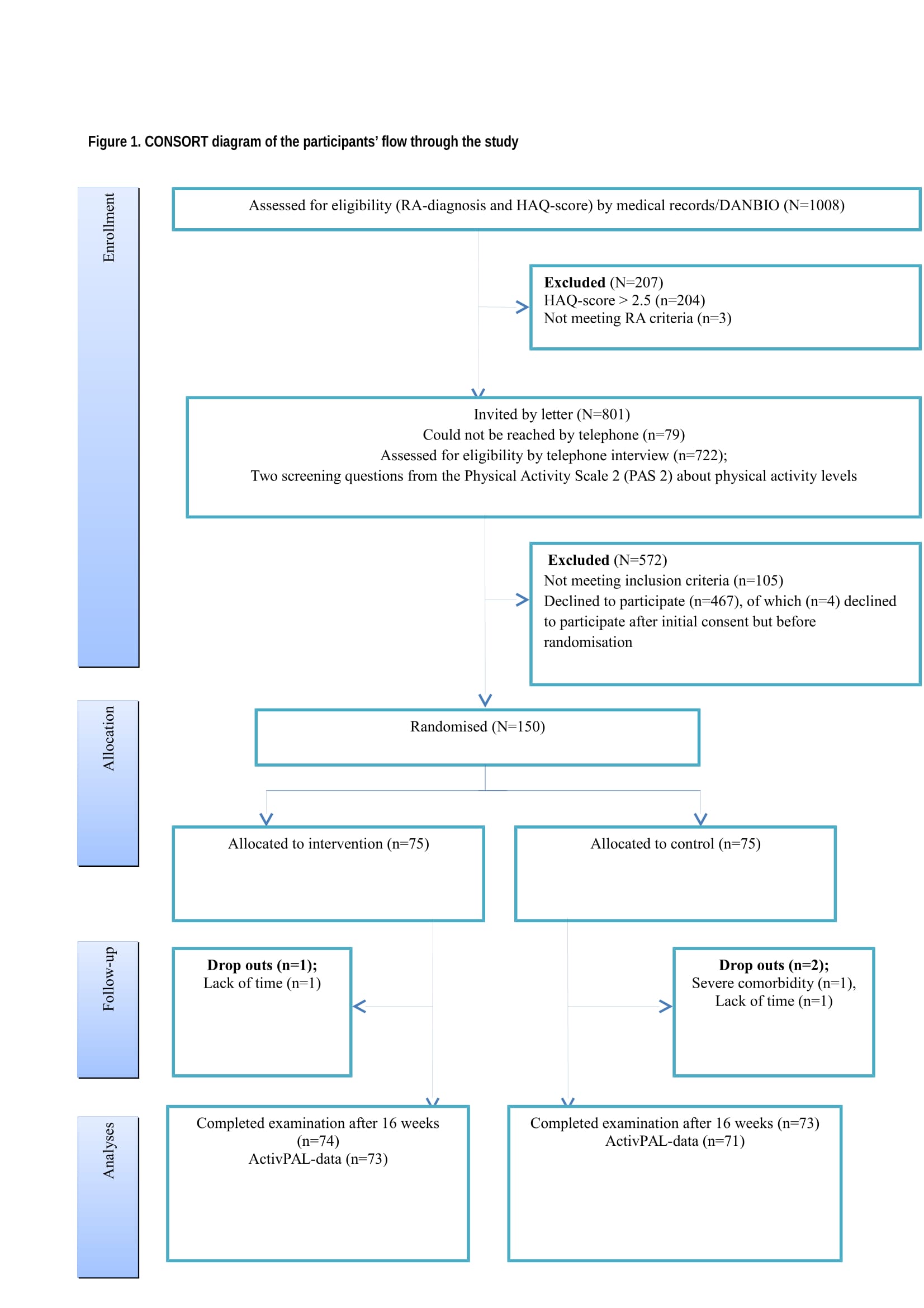

Supplement: Supplementary figure 1 [file annrheumdis-2016-210953supp002.jpg]

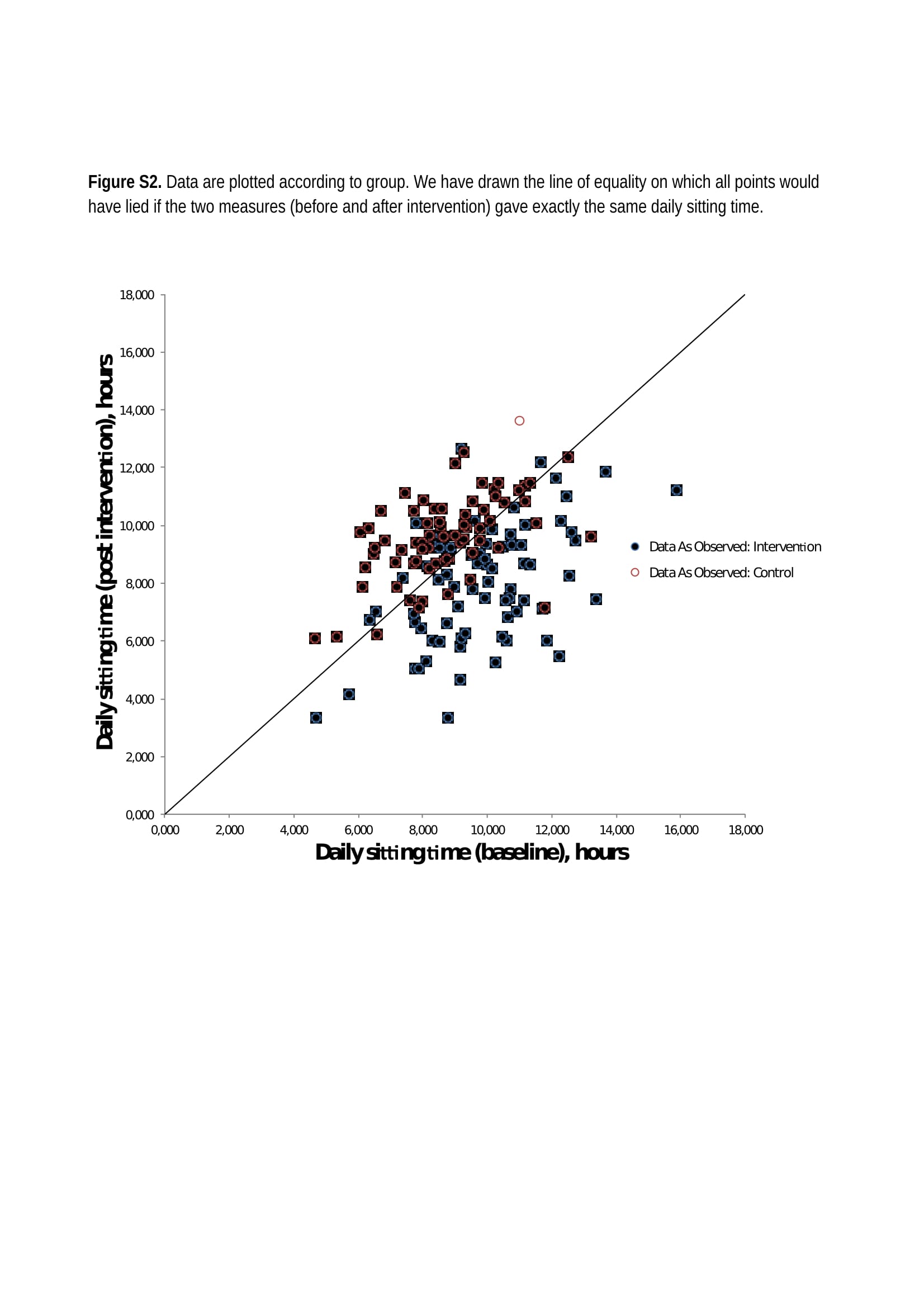

Supplement: Supplementary figure 2 [file annrheumdis-2016-210953supp003.jpg]
