# Supplementary material for: The efficacy of motivational counselling and SMS reminders on daily sitting time in patients with rheumatoid arthritis: a randomised controlled trial
Source: Ann Rheum Dis. 2017 Jun 5;76(9):1603–6. doi: 10.1136/annrheumdis-2016-210953 (PMC5561370; doi:10.1136/annrheumdis-2016-210953)
Supplement: Supplementary table 1 [file annrheumdis-2016-210953supp001.docx]

**Table S1**. Baseline characteristics of the participants by allocated group

| **Characteristic** | **Intervention group (N=75)** | **Control group**  **(N=75)** |
| --- | --- | --- |
| Women | 61 (81 %) | 60 (80 %) |
| Age (years)^a^ | 59.7 (10.7) | 59.5 (12.7) |
| Seasonal variation (included during winter) | 15 (11.3 %) | 17 (12.8 %) |
| Cohabiting | 53 (71 %) | 50 (66 %) |
| **Highest attained education**  Primary school  High school  Short to middle higher education  Long higher education (university degree) | 10 (13 %)  16 (22 %)  40 (54 %)  9 (12 %) | 11 (14 %)  14 (18 %)  39 (52 %)  11 (15 %) |
| **Occupation**  Unemployed  Employed full time  Employed part time  Age-related retirement  RA-related retirement | 4 (5 %)  12 (16 %)  18 (24 %)  38 (51 %)  3 (4 %) | 6 (8 %)  19 (25 %)  14 (19 %)  30 (40 %)  6 (8 %) |
| Smoking | 15 (20 %) | 14 (19 %) |
| Alcohol (drinks per week)^b^ | 1.0 (0.0,4.0) | 2.0 (1.0,5.0) |
| RA duration (years)^b^ | 12.0 (8.0,20.0) | 11.0 (7.0,20.0) |
| Medical treatment (Biologics) | 23 (31 %) | 21 (28 %) |
| **DAS-28^a^**  CRP^b^  Tender joints^b^  Swollen joints^b^  Global VAS^b^ | 3.1 (1.2)  6.0 (5.0, 11.0)  1.0 (0.0,2.0)  0.0 (0.0,1.0)  49.0 (28.0,66.0) | 3.2 (1.3)  5.0 (5.0, 10.0)  0.0 (0.0,1.0)  0.0 (0.0, 1.0)  25.0 (16.0,48.0) |
| Positive anti-CCP | 55 (73 %) | 59 (79 %) |
| Positive IgM-RF | 56 (75 %) | 61 (81 %) |
| Comorbidity | 59 (79 %) | 59 (79 %) |
| Self-reported moderate physical activity, hours/week^b^ | 2.0 (1.0,4.0) | 3.5 (2.0,6.0) |
| Self-reported vigorous physical activity, hours/week^b^ | 0.0 (0.0,0.5) | 0.3 (0.0,2.0) |
| Standing time (ActivPAL), hours/day^a^ | 4.0 (1.3) | 4.5 (1.4) |
| Stepping time (ActivPAL), hours/day^a^ | 1.6 (0.7) | 1.9 (0.7) |
| Daily sitting time (ActivPAL) hours/day^a^ | 9.8 (1.9) | 8.8 (1.7) |
| Breaks up of sitting time (ActivPAL), number/day^a^ | 53.0 (14.0) | 56.0 (17.0) |
| **Working participants**  Self-reported sitting at work, hours/day^b^ | 30 (40 %)  4.6 (2.0,6.0) | 33 (44 %)  5.0 (3.0,6.0) |
| Self-reported sitting time in leisure, hours/day^a^ | 5.3 (2.4) | 4.3 (2.0) |
| Physical function (HAQ)^b^ | 0.9 (0.5;1.4) | 0.5 (0.0,1.1) |
| **Fatigue (MFI)^a^**  General fatigue  Physical fatigue  Reduced activity  Reduced motivation  Mental fatigue | 13.5 (3.9)  14.0 (3.7)  13.1 (3.7)  10.0 (3.0)  10.6 (3.6) | 11.8 (4.2)  11.8 (3.7)  11.4 (4.0)  9.0 (2.8)  9.3 (3.9) |
| Fatigue (VAS)^b^ | 62.0 (31.0,76.0) | 30.0 (13.0,56.0) |
| Pain (VAS)^b^ | 41.0 (21.0,63.0) | 21.0 (11.0,33.0) |
| **HR-QoL (SF-36)^a^**  SF36-PCS  SF36-MCS | 35.3 (9.4)  50.9 (10.4) | 40.5 (10.5)  53.5 (9.3) |
| Self-efficacy (GSES)^a^ | 28.2 (5.9) | 31.5 (5.8) |
| **Lipids (mmol/L)**  Cholesterol (total)^a^  HDL^a^  LDL^a^  Triglyceride^b^ | 5.4 (1.1)  1.6 (0.5)  3.2 (0.9)  1.2(0.9,1.7) | 4.9 (1.0)  1.5 (0.4)  2.9 (0.9)  1.1 (0.8,1.7) |
| HbA1c (mmol/mol)^a,c^ | 6.0 (1.2) | 6.2 (1.3) |
| **Blood pressure (mmHg)^a^**  Systolic  Diastolic | 129.9 (17.7)  78.3 (10.0) | 131.5 (21.4)  77.7 (9.2) |
| Weight (Kg)^a^ | 74.4 (17.7) | 76.0 (16.6) |
| Waist circumference (cm)^a^ | 91.2 (14.8) | 92.9 (13.4) |
| BMI^a^ | 26.0 (5.5) | 26.8 (5.3) |
| Waist-hip-ratio^b^ | 0.9 (0.8,0.9) | 0.9 (0.8,0.9) |

Data are presented as N (%) unless otherwise stated

^a^ Mean (SD)

^b^ Median and interquartile range (Q1,Q3)

^c^ Participants were not fasting before measurement of HbA1c

Abbreviations; CRP, C-reactive protein; HAQ, Health Assessment Questionnaire; MFI, Multidimensional Fatigue Inventory; VAS, Visual Analogue Scale; PCS, Physical component Scale; MCS, Mental Component Scale; GSES, General Self-Efficacy Scale; HDL, High-density Lipoprotein cholesterol; LDL, Low-density Lipoprotein cholesterol.
